# Minocycline is a promising candidate as a combination therapy with caspofungin for drug-resistant *Candida*

**DOI:** 10.3389/fcimb.2024.1452497

**Published:** 2024-09-30

**Authors:** Kazuhiro Itoh, Hiroshi Tsutani, Yasuhiko Mitsuke, Hiromichi Iwasaki

**Affiliations:** ^1^ Department of Internal Medicine, NHO Awara National Hospital, Awara, Japan; ^2^ Division of Infection Control and Prevention, University of Fukui Hospital, Fukui, Japan

**Keywords:** minocycline, caspofungin, antifungal drug resistance, target of rapamycin proteins, *Candida*

## The rise in antifungal-resistant fungi

*Candida* spp. is the most common cause of fungal infection, and the emergence of drug resistance has become a significant concern in recent years. Notably, in 2009, *Candida auris*, a multidrug-resistant *Candida species*, was first identified from an ear discharge in a patient in Japan (Satoh et al., 2009). *Candida auris* is highly transmissible in healthcare settings and often leads to bloodstream infections, resulting in a greater fatality rate compared to other *Candida* spp (Lockhart et al., 2017; Jeffery-Smith et al., 2018; Spivak and Hanson, 2018).

## Currently used antifungal drugs and their drug-resistance mechanisms

The antifungal drugs currently utilized in clinical practice include azoles, polyenes, echinocandins, and flucytosine. In this section, we delineate the mechanisms of action and resistance for each.

Azoles inhibit the activity of cytochrome P450 sterol 14-demethylase, an enzyme that the *ERG11* gene produces. This impairs cell membrane integrity by blocking ergosterol production. Tolerance mechanisms include several strategies, such as mutations in the *ERG11* gene leading to lower drug affinity, increased *ERG11* expression, changes in the *ERG3* gene preventing harmful sterol buildup, and activation of drug efflux pumps resulting in reduced intracellular drug concentration (Ghannoum and Rice, 1999).

The polyenes’ mechanism of action involves damaging the plasma membrane by targeting ergosterol, whereas the resistance mechanism is primarily the production of low-affinity sterols via alterations in the sterol synthesis system (Ghannoum and Rice, 1999).

Echinocandins impede cell wall development by blocking the synthesis of (1→3)-β-D-glucan. When mutations happen in the *FKS1* or *FKS2* genes, they change the structure of (1→3)-β-D-glucan synthase, making it resistant to echinocandins (Ghannoum and Rice, 1999).

Flucytosine works by stopping pyrimidine metabolism and stopping fungal cells from making RNA, DNA, and proteins. The resistance mechanism occurs when there is a lack or decreased activity of uracil phosphoribosyltransferase, leading to the inability to create active metabolites (Ghannoum and Rice, 1999).

## Repurposing of existing antifungal drugs

Currently, there is a limited selection of antifungal drugs, with the most widely used being azoles, echinocandins, and polyenes. However, inherent obstacles have hindered the advancement of novel antifungal drugs. Specifically, fungi, which are eukaryotes like humans, pose a challenge in developing compounds that exhibit high efficacy against fungal pathogens while maintaining low toxicity to host cells. Furthermore, the lack of significant financial incentives for pharmaceutical companies has contributed to this area’s lack of progress (Robbins et al., 2016). An alternative approach that does not depend on the creation of novel drugs is to repurpose existing drugs by combining them. Research has demonstrated the effectiveness of combining antifungal drugs in treating *Cryptococcus neoformans* and *Scedosporium* spp (Brennan-Krohn et al., 2021). However, the limited types of drugs it can combine with other antifungal drugs have restricted its use so far.

## Synergistic effects of combining caspofungin and a target of rapamycin (TOR) inhibitor against drug-resistant *Candida*

Lefranc et al. suggested the synergistic antifungal effects of rapamycin and caspofungin against drug-resistant strains of *Candida* (Lefranc et al., 2024). The authors determined that the TOR pathway was able to recognize the cell wall stress caused by antifungal drugs and had a role in the vulnerability of *Candida* species to echinocandins. Furthermore, they acknowledged the potential of rapamycin, which is an immunosuppressant, as an enhancer when combined with existing antifungal drugs. The findings of their research provided new perspectives on overcoming resistance mechanisms in *Candida* species. Nevertheless, in the context of medical practice, we believe that minocycline, a type of tetracycline antibiotic, is better suited as a combination therapy with caspofungin than rapamycin.

## Minocycline as a promising candidate for combination therapy with caspofungin against drug-resistant *Candida*

With regard to the factors that support our postulate, first, Lefranc et al. asserted that the impact of rapamycin on *Candida* spp. was indicative of crosstalk between the TOR signaling pathway and the cell wall integrity (CWI) pathway (Lefranc et al., 2024). Given that this is the mechanism by which the synergistic action of rapamycin and caspofungin occurs, this poses a new issue. It has been shown that caspofungin resistance is due to inhibition by rapamycin of the TOR pathway, which then activates the CWI pathway (Torres et al., 2002). However, a different explanation of the molecular mechanism via the TOR pathway would be required in order to account for the ability of rapamycin to enhance the effects of caspofungin. Meanwhile, a previous study has demonstrated that minocycline has inhibitory effects on TOR (Qiao et al., 2023). Minocycline is a tetracycline antibiotic with an antibacterial mechanism of action that involves binding to the 30S ribosomal subunit, which inhibits the transport of charged tRNAs that carry amino acids. This impedes protein chain elongation and cellular protein production. Minocycline has efficacy against both Gram-positive and Gram-negative bacteria, making it a suitable therapeutic option for the treatment of pneumonia, bloodstream infections, as well as skin infections (Nazarian and Akhondi, 2024). The study by Lefranc et al. confirmed that rapamycin, which is a TOR inhibitor, increased the susceptibility of the *Candida* spp. to caspofungin (Lefranc et al., 2024). In fungal cells, TOR facilitates Sit4 and Tap42 binding while inhibiting cell membrane stress. Thus, it is plausible that the cellular membrane stress is a result of the inhibitory impact of minocycline on TOR (Torres et al., 2002). Therefore, it is conceivable that minocycline could potentially enhance the susceptibility of *Candida* spp. to caspofungin. Furthermore, another study has verified the synergistic impact of combining caspofungin and minocycline for combatting the multidrug-resistant *Candida auris* (Brennan-Krohn et al., 2021). The authors of that study suggested that caspofungin hinders the formation of the cell wall, thereby compromising its integrity. At the same time, minocycline enters the cell and inhibits protein synthesis in the ribosomes of yeast-like fungi. The combination of amphotericin B and rifampicin, as well as the combination of amphotericin B and minocycline, was investigated as a treatment for *Candida auris*. Rifampicin hinders the activity of RNA polymerase in yeast, whereas minocycline impedes protein synthesis in yeast ribosomes. Furthermore, research suggests that amphotericin B acts as a “sterol sponge,” causing cell death by eliminating ergosterol from the lipid bilayer as large aggregates form outside the membrane. As a result, it is believed that the combination of these drugs has a synergistic effect because amphotericin B exerts a sub-lethal activity that creates pores in fungal cells, enabling the penetration of the antimicrobial agents used in combination (Brennan-Krohn et al., 2021). The checkerboard array assay revealed that the minimum inhibitory concentrations (MICs) of caspofungin and minocycline against *Candida auris* were 0.125-2 μg/mL and >64 μg/mL, respectively. When used together, the MICs of caspofungin and minocycline were 0.031-0.125 μg/mL and 4-16 μg/mL, respectively. The fractional inhibitory concentration index ranged from 0.094 to 0.375, indicating a synergistic effect (Brennan-Krohn et al., 2021).

Second, due to its intrinsic immunosuppressive properties, rapamycin has the potential to worsen infections as an adverse effect. A systematic review of rapamycin revealed that infections were a common adverse event, with a limited number of severe cases. However, there have also been reported cases of treatment-related fatalities attributed to pneumonia and septic shock (Lee et al., 2024).

Third, there are difficulties with drug interactions. The coadministration of rapamycin with the echinocandin antifungal micafungin was reported to lead to a 21% increase in the area under the curve for rapamycin (Wiederhold et al., 2008). Thus, concurrent use of caspofungin and rapamycin could potentially lead to an elevated risk of rapamycin toxicity.

## Host immune system modulation effects of antimicrobial agents

In previous research, we found that both caspofungin and minocycline suppress the inflammatory signaling pathways in the host’s immune system when they are activated by lipopolysaccharide from bacteria and zymosan from yeast-like fungi (Tai et al., 2013; Itoh et al., 2021). Candidemia is characterized by an excessive production of inflammatory cytokines, which leads to organ dysfunction and vascular injury due to the overproduction of these cytokines (Presterl et al., 1999). Spleen tyrosine kinase (Syk) is crucial in the immunological response of host immune cells to fungal infections. In a zymosan-induced sepsis model, the use of Syk inhibitors resulted in improvement in shock states (Unsal et al., 2018). These Syk-dependent pathways are blocked by caspofungin (Itoh et al., 2021), while the inhibitor of nuclear factor-κB kinase (IKK) α/β pathway of the Toll-like receptor 4 (TLR4) pathway is blocked by minocycline (Tai et al., 2013). Both of these drugs efficiently suppress the overproduction of inflammatory cytokines and chemokines by the immune cells of the host, thereby serving as a protective mechanism against sepsis. When it comes to clinical application, the immunomodulatory effects, akin to a shield for humans, are crucial.

## Conclusion

**Figure 1:**
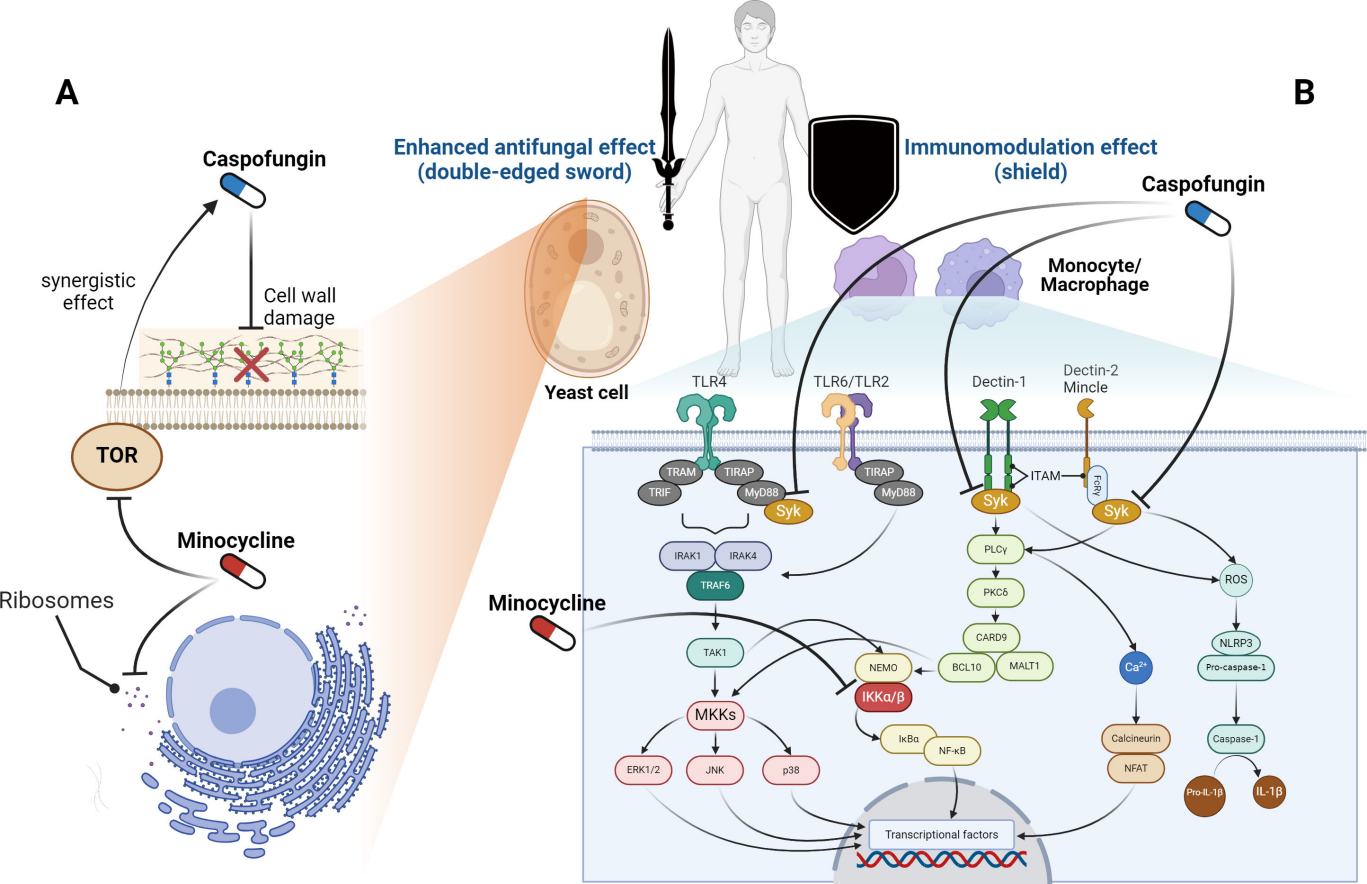
Sites of action for caspofungin and minocycline. **(A)** In yeast-like fungi, caspofungin inhibits β-glucan synthase and targets the cell wall, whereas minocycline works on ribosomes and suppresses protein synthesis inside the cell. Furthermore, minocycline inhibits the target of rapamycin (TOR) and enhances the effectiveness of caspofungin. **(B)** Pattern recognition receptors on the membranes of host immune cells recognize fungi and transmit signals inside the cells. Syk is located upstream of these signaling pathways, while caspofungin inhibits Syk-dependent pathways. Furthermore, in these signaling pathways, minocycline inhibits the activity of the inhibitor of nuclear factor-κB kinase (IKK) α/β. BCL10, B-cell lymphoma 10; CARD9, caspase recruitment domain-containing protein 9; ERK, extracellular signal-regulated kinase; FcγR, Fc gamma receptor; IκBα, inhibitor of nuclear factor kappa-B kinase alpha; IL-1β, interleukin 1 beta; IRAK, IL-1 receptor-related kinase; ITAM, immunoreceptor tyrosine-based activation motif; JNK, c-Jun N-terminal kinase; MALT1, mucosa-associated lymphoid tissue lymphoma translocation protein 1; MKK, mitogen-activated protein kinase kinase; MyD88, myeloid differentiation factor 88; NEMO, nuclear factor-kappa B essential modulator; NFAT, nuclear factor of activated T-cells; NF-κB, nuclear factor kappa-B; PLCγ, phospholipase-C gamma; PKCδ, protein kinase-C delta; ROS, reactive oxygen species; Syk, spleen tyrosine kinase; TAK1, transforming growth factor-β-activated kinase 1; TIR, Toll/interleukin-1 receptor; TIRAP, TIR domain-containing adaptor protein; TLR, toll-like receptor; TRAM, TRIF-related adaptor molecule; TRIF, TIR domain-containing adaptor protein inducing interferon-β. The figure was created using BioRender (https://biorender.com/).

Considering the above, we conclude that minocycline, when combined with caspofungin, is a more suitable option than rapamycin for clinical usage. As a double-edged sword against *Candida*, minocycline enhances the antifungal activity of caspofungin, while also modulating overactive immune systems as a shield in humans (**Figure 1**). Specifically, in yeast-like fungi, caspofungin inhibits β-glucan synthase from working, which damages the cell wall. Minocycline inhibits the ribosomes inside the cell from making proteins. Also, minocycline inhibits the TOR protein from working, which makes caspofungin more effective against fungi through a synergistic effect. On the other hand, the cell membrane of host immune cells has pattern recognition receptors that identify fungi and send signals to the cell. Syk initiates multiple signal pathways and regulates component activation in downstream molecules. The caspofungin suppresses the Syk-dependent pathways, and the minocycline suppresses IKK α/β, which is further down these signaling pathways. These findings imply that this usage has the potential to treat *Candida* infections, including those from drug-resistant *Candida* strains.
